# Person-Centered Long-Term Care Standards: Research, Education, and Tool Development

**DOI:** 10.1093/geroni/igaf122.552

**Published:** 2025-12-31

**Authors:** Tara Cortes

## Abstract

The Mayer-Rothschild Foundation (TM-RF) was founded to further Dr. Robert N. Mayer’s vision of person-centered long-term care. The Foundation has generously funded this research and scholarship. The ultimate goal of the TM-RF Designation of Excellence in Person-Centered Long-Term Care process is to establish standards and processes that can be used to recognize long-term care settings which implement best practices in person-centered care. The Designation of Excellence is the result of several years of research, testing, and evaluation by innovators and early adopters across the long-term care sector. In this symposium, the HIGN team at NYU Meyers presents their endeavor in this important work, building on prior work completed by the University of Maine during the first two years of this multi-year project. This symposium describes how qualitative and quantitative data collected from multiple sites selected for geographical and population diversity were used to validate domains and concepts that can be used to describe person-centered care. The session also describes the development of an organizational assessment tool using these domains and concepts.

